# Nitrous Oxide “Whippet” Induced Subacute Combined Degeneration of the Spinal Cord: A Case Report

**DOI:** 10.5811/cpcem.64776

**Published:** 2026-10-30

**Authors:** Joshua Kern, Carolyn Shanks

**Affiliations:** University of Texas-Southwestern, Department of Emergency Medicine, Division of Medical Toxicology, Dallas, Texas

**Keywords:** subacute combined degeneration, vitamin B12 deficiency, nitrous oxide, myelopathy, emergency medicine

## Abstract

**Introduction:**

Subacute combined degeneration (SCD) is an uncommon neurologic complication of vitamin B12 deficiency characterized by demyelination of the dorsal and lateral columns of the spinal cord, resulting in weakness, paresthesias, sensory deficits, and gait ataxia. Recreational nitrous oxide inhalation has emerged as an important cause of functional vitamin B12 deficiency and may precipitate SCD, particularly in otherwise healthy individuals.

**Case Report:**

We report a case of nitrous oxide-associated SCD in a patient who presented to the emergency department with progressive neurologic symptoms. This case illustrates a rare but increasingly recognized complication of recreational nitrous oxide use.

**Conclusion:**

Nitrous oxide-induced SCD should be considered in patients presenting with subacute weakness, paresthesia, and ataxia, particularly when there is a history of recreational inhalant use. Early recognition and treatment with intramuscular vitamin B12 replacement, together with cessation of nitrous oxide exposure, are essential to optimize neurologic recovery.

## INTRODUCTION

Inhaled nitrous oxide (N_2_O) has emerged as an increasingly prevalent substance for recreational use. Historically, it was used in medical settings (including dentistry) for its quick anesthetic effects. Commonly known as “laughing gas,” N_2_O produces a brief period (approximately 20 seconds) of euphoria, contributing to both its therapeutic appeal and its misuse.^1^ Beyond healthcare, N_2_O is also a popular food additive, functioning as a propellant for certain foods, including whipped cream.^1^ Recreational use of N_2_O is not a recent phenomenon. “Nitrous oxide frolics” date back to the early 1800s, just years after its initial discovery. Abuse was particularly common among medical professionals who used N_2_O in their practices.^2^

Since 2000, recreational N_2_O use has become more widespread.^3^ It is commonly obtained from steel cartridges filled with compressed N_2_O used for preparation of whipped cream. When used in this manner, it is colloquially referred to as whippets, nitro, nangs, or balloons. These N_2_O canisters are easy and legal to obtain, which could explain the popularity and growing use, especially among the younger population.^3^ The 2019 Global Drug Survey, which gathers data from self-selected drug users from over 30 countries, found that 91% of those surveyed (*n* = 123,814) had used N_2_O at least once in their lifetime.^4^

When used in a controlled hospital setting, N_2_O is remarkably safe.^5^ Vitamin B12 deficiency is rare in healthy individuals, as hepatic stores of vitamin B12 are typically sufficient to last years. However, certain populations are at increased risk, including those with decreased dietary intake of vitamin B12 (such as those following vegan diets) and those with malabsorptive issues such as pernicious anemia, atrophic gastritis, or ileal resection.^5^

Nitrous oxide can cause vitamin B12 (cyanocobalamin) deficiency secondary to oxidation and inactivation of the vitamin B12 enzyme. Nitrous oxide oxidizes a cobalt ion on the vitamin B12 coenzyme, which converts it from an active monovalent form (CO^+^) to an inactive divalent form (CO^2+^); this irreversibly inhibits its enzymatic activity. Furthermore, the cobalt moiety of vitamin B12 functions as an essential methyl carrier in the formation of methionine from homocysteine and 5-methyltetrahydrofolate. Inactivation of this pathway impairs methionine synthesis, leading to downstream inhibition of DNA synthesis and myelin production, which underlies the hematologic and neurologic manifestations of N_2_O toxicity^6^ (Figure).

Subacute combined degeneration (SCD) of the spinal cord often presents with progressively worsening neurologic deficits, including impaired vibratory sense and proprioception, ataxia, sensory loss, weakness, and spastic quadriparesis. These manifestations are likely secondary to demyelinating effects on the lateral corticospinal tracts and dorsal column of the spinal cord.^8^ Cognitive decline can also be appreciated, which is often reversible with timely recognition and treatment.^8^

Recreational use of N_2_O can also cause hematologic effects and bone marrow suppression, secondary to the effects of vitamin B12 deficiency and impaired methionine synthesis.^6^ Laboratory studies often reveal a macrocytic anemia with elevated homocysteine and methylmalonic acid (MMA) levels.^8^ Magnetic resonance imaging (MRI) of the entire spine is recommended when SCD is suspected, which can show characteristic T2 hyperintensity of the posterior (dorsal) columns in up to 68% of affected patients.^9^ Despite common clinical involvement of the lateral spinal cord tracts, corresponding radiographic abnormalities in this region are uncommon.^8^

The use of N_2_O has been linked to severe neurologic injury and death. However, these outcomes are thought to result predominantly from hypoxic and asphyxiation during inhalation, rather than secondary to direct neurotoxic effects of N_2_O.^10^

*CPC-EM Capsule*

What do we already know about this clinical entity?

*Recreational nitrous oxide use can inactivate vitamin B12, causing subacute combined degeneration with progressive weakness, sensory loss, and ataxia.*

What makes this presentation of disease reportable?

*Severe neurologic dysfunction with unrevealing spinal magnetic resonance imaging (MRI), absent macrocytosis, and bilateral pulmonary emboli highlights an easily missed diagnosis.*

What is the major learning point?

*Nitrous oxide-induced myelopathy can occur without characteristic MRI findings or macrocytosis; exposure history and biochemical testing are key to diagnosis.*

How might this improve emergency medicine practice?

*Targeted questioning about nitrous oxide use and early B12 testing can expedite treatment and cessation counseling, potentially reducing lasting disability.*

## CASE REPORT

A 27-year-old man with a history of prior substance use disorder, including marijuana and N_2_O (“whippets”), presented to the emergency department (ED) after his father requested a welfare check by emergency medical services. According to his father, the patient had experienced 3 days of progressively worsening mentation and had become bedridden. On presentation, he was unable to provide a meaningful history secondary to his altered mentation, and he reported only left thigh pain. Additional history obtained from the patient’s father revealed that his son had recently been admitted to an outside hospital due to a subacute inability to walk. During that admission, he had an extensive workup including MRI and lumbar puncture, but no definitive diagnosis was made.

Initial vital signs were notable for the following: tachycardia (heart rate, 108 beats per minute); hyperthermia (37.8 °C); hypotension (78/50 mm Hg); and hypoxemia with SpO_2_ in the upper 80% range on room air. Examination was notable for an ill-appearing patient, covered in feces and urine. He was oriented only to self and year but was otherwise unable to answer questions. Neurologic examination demonstrated marked bilateral lower extremity weakness; sensation was difficult to assess secondary to his neurologic status. No clonus or rigidity was appreciated on exam. Lower extremity reflexes were brisk. His abdomen was distended and tender in the lower region. He had 2+ pitting edema in the bilateral lower extremities.

A broad workup was initiated. Point-of-care bladder ultrasonography revealed significant urinary retention with greater than 2 L of fluid in his bladder, for which a Foley catheter was placed. Imaging studies were obtained including computed tomography (CT) of the head, CT of the thoracic spine, CT of the lumbar spine, CT of the abdomen and pelvis, and CT angiography (CTA) of the chest. Findings on the CTA of the chest revealed acute pulmonary emboli in the bilateral lungs, as well as a consolidation in the left lower lung lobe. Computed tomography of the head revealed mild generalized volume loss, advanced for the patient’s age. An MRI lumbar spine demonstrated no acute findings.

Laboratory workup revealed a mild normocytic anemia with a hemoglobin of 12.3g/dL (reference range, 13.2–16.9 g/dL) and a mean corpuscular volume of 88.9 fL (80–100 fL). There was no leukocytosis or thrombocytopenia. Serum creatinine was elevated to 1.73 mg/dL (0.67–1.17 mg/dL), with a creatine kinase elevation to 1,888 U/L (39–308 U/L), which eventually peaked at 2,827 U/L. Thyroid-stimulating hormone was within normal limits. Urine toxicology was positive for cannabinoid use, while the serum ethanol level was undetectable. Further evaluation revealed an undetectable vitamin B12 level. A subsequent homocysteine and methylmalonic acid were obtained; homocysteine and MMA levels were markedly elevated at 60.6 mmol/L (5–15 μmol/L) and 15.93 mmol/L (< 0.04 μmol/L), respectively (Table).

Resuscitative measures were initiated, including intravenous fluids and empiric antibiotic therapy. A heparin drip was started. The patient was subsequently admitted to the hospital. The neurology team agreed that this case was suspicious for SCD secondary to whippet use. Additional MRI studies of the brain, cervical spine, and thoracic spine were obtained; however, no definitive radiographic abnormalities were identified (Image 1 and 2).

The patient was started on aggressive vitamin B12 repletion, starting with intramuscular (IM) vitamin B12 1,000 mcg daily for 7 days, followed by IM vitamin B12 1,000 mcg weekly for 4 weeks. Subsequently, he was transitioned to oral vitamin B12 1,000 mcg daily for ongoing maintenance therapy. Over the course of his hospitalization, the patient demonstrated gradual neurologic improvement, and he was discharged to an inpatient rehabilitation facility for continued recovery.

## DISCUSSION

Recreational N_2_O use and its neurologic complications are increasingly recognized.^4^ Many of the neurologic and hematologic complications arise from deficiencies in vitamin B12, which are induced from N_2_O use. Diagnosing use in the ED can be challenging, as there are no readily available screening tests to confirm exposure. Additionally, patients may not disclose their use, or they may be unable to provide an accurate history due to altered mental status.

Neuroimaging, particularly MRI, can support the diagnosis of N_2_O-induced SCD. Characteristic findings include T2-weighted hyperintensities in the posterior and lateral columns of the spinal cord, reflecting involvement of the dorsal columns and corticospinal tracts.^7^,^11^ These abnormalities are most commonly seen in the cervical region, followed by the thoracic region.^7^,^11^ Brain MRI is often unrevealing, with no abnormalities identified in most cases.^11^ Importantly, the absence of radiographic findings does not exclude the diagnosis; characteristic imaging findings are only seen in approximately 68% to 78% of diagnosed cases.^9^

Certain laboratory studies can further support the diagnosis. Patients will often have a macrocytic anemia, although this finding is not universally present. Patients will also likely have low serum vitamin B12 levels, as well as an elevated homocysteine and methylmalonic level.^8^ Importantly, vitamin B12 levels may be falsely normal or even elevated in some SCD patients. Obtaining homocysteine and methylmalonic levels can be helpful for diagnosis when clinical suspicion remains high.^8^

Our patient also presented with a concurrent diagnosis of acute pulmonary embolism. Although this may initially appear to have been unrelated, a potential mechanistic link exists through the prothrombotic effects of elevated homocysteine levels. It is well-established that elevated homocysteine levels are associated with thrombotic diseases through a variety of mechanisms including endothelial dysfunction, oxidative stress, impaired anticoagulant pathways, and promotion of coagulation activation.^15^

The primary treatment for SCD secondary to N_2_O use is aggressive vitamin B12 replacement. Although recommendations vary, it is reasonable to start with 1,000–2,000 mcg of daily IM vitamin B12 for 1 week, followed by 1,000 mcg IM vitamin B12 weekly for 4 to 9 weeks.^6^,^12^ After this initial repletion, patients should continue maintenance therapy with daily oral administration of 1,000–2,000 mcg of B12 supplementation until resolution of symptoms.^6^,^12^ Prognosis is generally favorable with prompt recognition and treatment, although the degree of neurologic recovery is variable. A 2022 review of 96 patients found that approximately 75% experienced significant improvement in their neurologic symptoms after appropriate vitamin B12 supplementation.^13^ Another review found that 86% of patients had significant improvement, but only 14% achieved complete resolution of their symptoms.^14^

Several factors have been associated with improved neurologic outcomes, including younger age at presentation (< 50 years), shorter duration of symptoms, absence of sensory deficits, absence of a positive Romberg sign, absence of a positive Babinksi sign, involvement of fewer spinal segments on MRI (fewer than 7), presence of spinal cord edema, and absence of spinal cord atrophy.^14^

Additional supplementation with methionine may be beneficial, as methionine is a direct substrate for methionine synthase.^14^ In addition to medical therapy, physical therapy and rehabilitation plays an essential role in recovery.^12^ Finally, sustained abstinence from N_2_O exposure is also critical to prevent recurrence of neurologic injury.

## CONCLUSION

This case report details the diagnosis, management, and prognosis of subacute combined degeneration secondary to recreational use of nitrous oxide. As recreational N_2_O becomes more prevalent, clinicians must recognize this physiology and maintain a high index of suspicion in patients with unexplained neurologic symptoms. Early recognition and intervention are essential to preventing long-term neurologic sequelae.

## Figures and Tables

**Figure.**
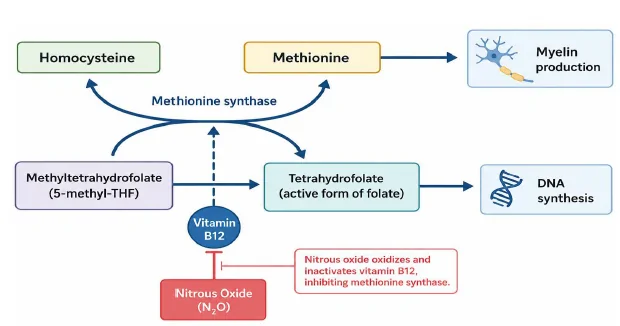
Depiction of vitamin B12 biochemistry.

**Image 1:**
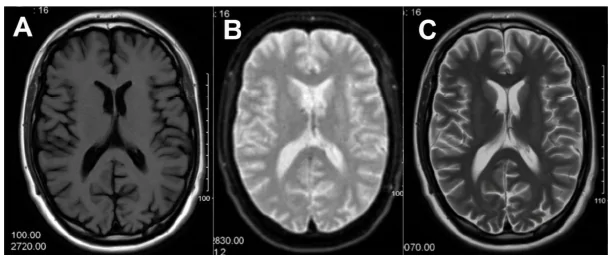
Magnetic resonance imaging of the brain: (**A**) T1 flair; (**B**) diffusion-weighted imaging; (**C**) T2 blade. No acute abnormalities appreciated.

**Image 2:**
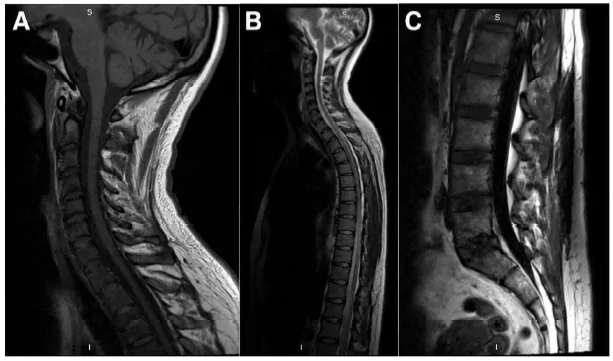
Magnetic resonance imaging: (**A**) cervical, (B) thoracic, and (**C**) lumbar. No acute abnormalities appreciated. Magnetic resonance imaging did not reveal the characteristic T2 hyperintensity of the posterior (dorsal) columns usually seen in patients with subacute combined degeneration.

**Table:** Initial laboratory studies in a patient presenting with altered mental status.

Laboratory value	Value on admission	Reference value
Hemoglobin	12.3 (g/dL)	13.2–16.9 (g/dL)
Mean corpuscular volume	88.9 (fL)	80–100 (fL)
Creatinine	1.73 (mg/dL)	0.67–1.17 (mg/dL)
Creatinine kinase	1,888 (U/L)	39–308 (U/L)
Vitamin B12	< 150 (pg/mL)	180–914 (pg/mL)
Homocysteine	60.6 (μmol/L)	5–15 (μmol/L)
Methylmalonic acid	15.93 (μmol/L)	0–0.04 (μmol/L)

*dL*, deciliter; *fL*, ; *g*, grams; *L*, liter; *mg*, milligram; *mL*, mililiter; *μmol*, micromoles; *pg*, picogams; *U*, units.
